# Endovascular abdominal aortic aneurysm repair in a patient with previous history of simultaneous orthotopic liver kidney transplantation

**DOI:** 10.1093/jscr/rjab332

**Published:** 2021-08-24

**Authors:** Javad Salimi, Ali Jafarian, Mohamad Behzadi, Afsaneh Nejat, Nasir Fakhar

**Affiliations:** Department of Surgery, Sina Hospital, Tehran University of Medical Sciences, Tehran, Iran; Department of Surgery, Imam Khomeini Hospital, Tehran University of Medical Sciences, Tehran, Iran; Department of Surgery, Faculty of Medicine, Tehran University of Medical Sciences, Tehran, Iran; Department of Surgery, Faculty of Medicine, Tehran University of Medical Sciences, Tehran, Iran; Department of Surgery, Shariati Hospital, Tehran University of Medical Sciences, Tehran, Iran

## Abstract

Management of abdominal aortic aneurysms (AAA) tends to be an issue in patients with a previous history of abdominal transplantation surgeries. Open surgery poses the risk of ischemia to the grafted tissue. Additionally, these patients have comorbidities that make them unable to endure such procedures. As a result, endovascular repair is becoming the accepted procedure in the transplanted population. Herein, we describe a patient with a previous history of simultaneous orthotopic liver-kidney transplantation who successfully underwent EVAR for AAA correction.

## INTRODUCTION

With the increasing number of successful abdominal organ transplantations, the transplanted population grows older; therefore, age-related cardiovascular comorbidities make them prone to vascular pathologies such as aneurysms. Managing aneurysms in the transplanted population is vitally important, as aneurysms tend to expand and rupture faster. Open repair and endovascular aortic repair (EVAR) are both surgical options; however, new studies suggest that EVAR is a safer choice in transplanted patients. In this report, we detail a case of abdominal aortic aneurysm in a patient with previous simultaneous orthotopic liver-kidney transplantation who was successfully treated with EVAR.

## CASE

**Figure 1:**
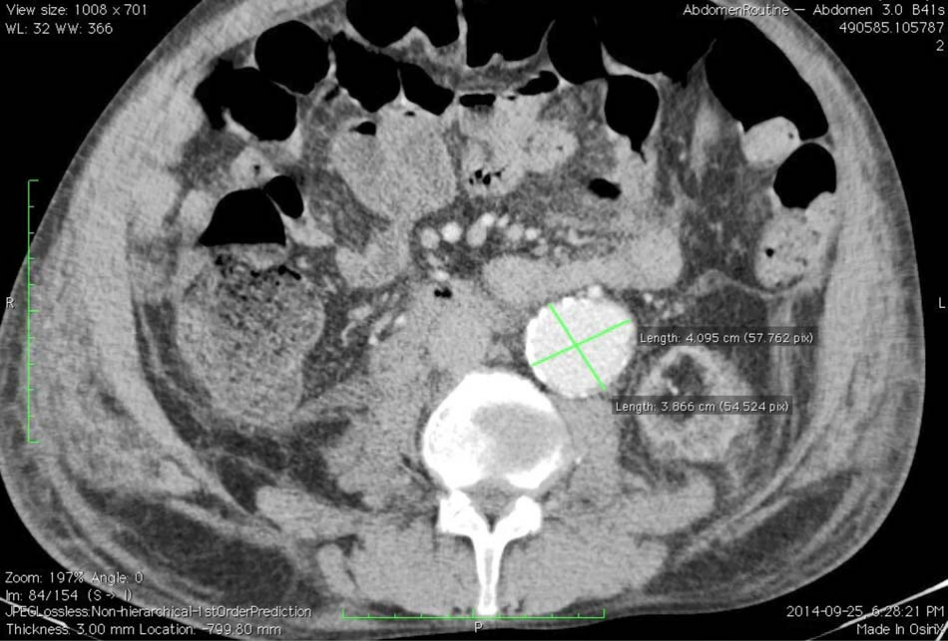
Pre-operative CT scan.

A 59-year-old male presented with end-stage liver disease secondary to hepatitis C and end-stage renal disease. On CT scan, an abdominal aortic aneurysm was incidentally found with a diameter of 4^*^3.8 cm (Fig. 1).

**Figure 2:**
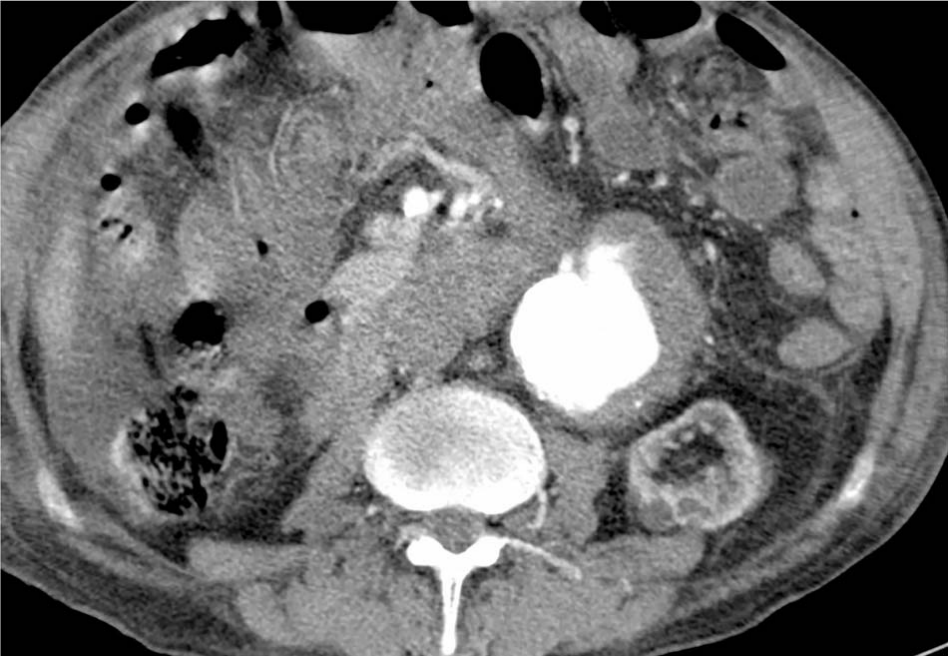
Post-operative contrast-enhanced CT scan, late arterial phase.

Given the rapid decompensation in the patient, a simultaneous liver-kidney transplant was proposed, but because the accepted diameter of aneurysm for surgical management is 5.5 cm and higher [1], it was decided to closely follow-up with the patient post-transplantation. The liver was transplanted using the piggy-back technique, inferior vena cava was anastomosed side-to-side, and portal veins and hepatic arteries were anastomosed end-to-end. The donor kidney was placed in the right iliac fossa with anastomosis of the right internal iliac artery and vein in an end-to-end fashion. The patient then had an uncomplicated post-operation course and was discharged with an immunosuppressive regimen. One month later, the patient came to the emergency room with severe abdominal pain. To rule out possible aneurysm rupture, an emergent CT scan with contrast was performed; however, the findings were compatible with an infrarenal aortic aneurysm with eccentric mural thrombosis. In addition, the aneurysm had grown in size and had a diameter of 5.2^*^6.5 (Fig. 2).

**Figure 3:**
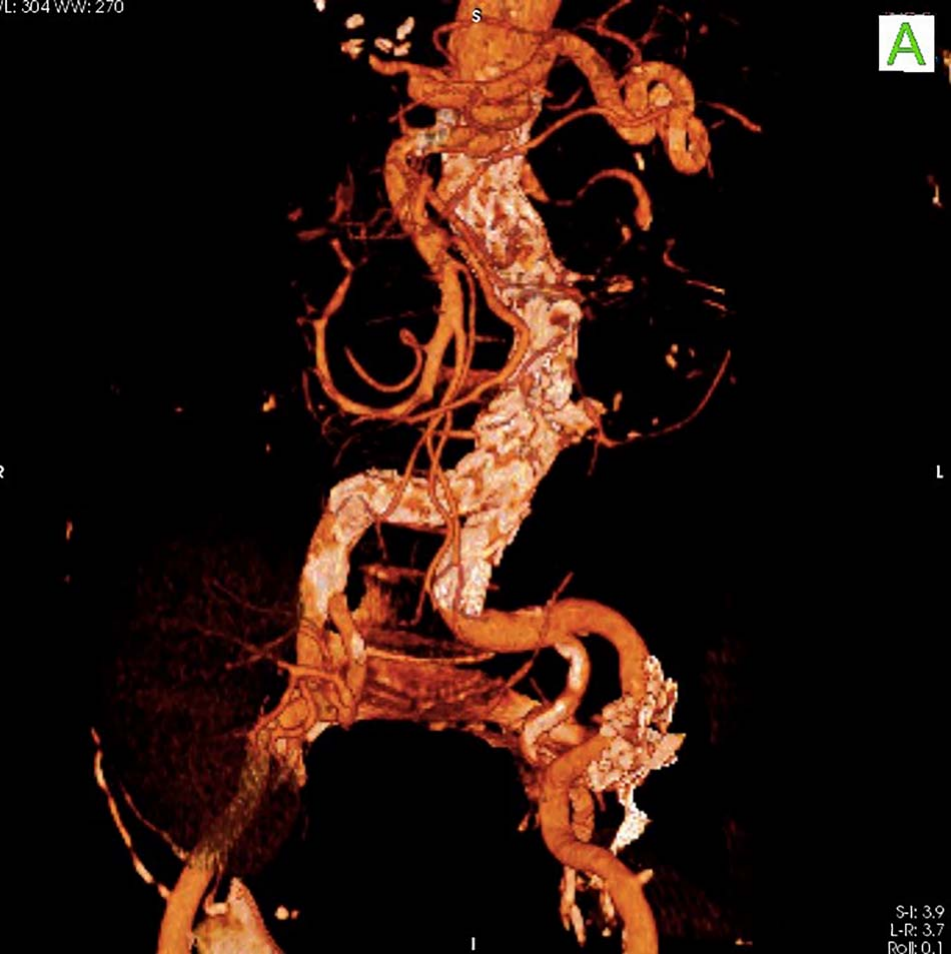
Follow-up CT angiography.

To minimize the risk of injury to renal allograft and avoid facing a hostile-abdomen, the patient was proposed to EVAR. Prior to intervention, upon contrast injection, the aneurysm and the corresponding clot containing the leakage were visualized (Video 1). A bifurcated endograft (Cook Zenith LP) was used and the main body was introduced through the left common femoral artery to reduce the duration of ischemia. The contralateral gate was cannulated and Lunderquist wire was advanced. The right-sided sheath was withdrawn up to the common femoral artery and the ipsilateral limb was deployed (Video 2). The post-operation course was uncomplicated with normal levels of serum creatinine. The patient was discharged 2 days later. One month later, a follow-up CT angiography was performed that demonstrated successful endovascular stent graft placement (Fig. 3).

## DISCUSSION

Previous studies support the claim that there is no significant difference in the incidence of AAA formation between the general population and abdominal organ transplant recipients [2]. However, rapid expansion rate and a consequently higher risk of rupture in the transplant population necessitate vigorous surveillance and follow-up [3].

Englesbe *et al.* studied 1557 heart, kidney and liver transplant recipients, of which 18 were diagnosed with AAA. The mean expansion rate before and after transplantation was 0.46 and 1.0 cm/year, respectively, similar for heart, kidney and liver transplants [4]. The data indicates that a mutual factor must explain the aggressive behavior of AAA in these patients. Immunosuppressive agents have been theorized to cause rapid AAA expansion through an unknown mechanism [5]. Additionally, other factors can contribute to higher rates of expansion in liver transplants, such as hypertension due to increased preload after removal of the cirrhotic liver and an improvement in the liver’s function to synthesize cholesterol [6]. The data affirm that AAA management is more crucial in these patients and meticulous observation is warranted.

Repairing AAA in a liver-kidney transplant patient brings its own set of challenges. The allograft kidney is a susceptible tissue considering a lack of collateral blood supply and denervation that causes disturbance in blood flow regulation. The most important challenge is preserving circulation to the transplanted kidney during the procedure.

Another challenge is facing a ‘hostile abdomen’ due to previous hepatic transplant surgery. Furthermore, many transplant patients suffer from cardiovascular comorbidities that need to be taken into account when choosing the type of procedure [7, 8].

The management of AAA can be categorized into open repair surgery with or without prophylactic measures to reduce the risk of renal ischemia and EVAR.

Different techniques have been introduced to reduce the risk of ischemia during open surgery repairs, such as axillofemoral bypass, aortoiliac bypass, femoral artery to femoral vein bypass with pump oxygenators, local allograft cooling, mannitol and fluid loading [7, 9]. However, none has proved to have any more advantage than a ‘clamp-and-go’ strategy [10].

EVAR helps avoid clamping the aorta, therefore, decrease the risk of ischemia and injury to renal allograft and not submit a previously operated patient to another laparotomy. It is minimally invasive, not requiring general anesthesia, with a shorter duration and a faster recovery [11]. Like any other procedure, EVAR carries its limitations and complications. The use of EVAR necessitates appropriate anatomy. Neck diameter, the shape of the aorta, the presence of calcification, thrombus and tortuosity should be considered. Other important criteria include the diameter of the access vessels and the presence of aneurysms in iliac arteries [12]. Contrast-induced nephropathy is a major concern in a kidney transplant patient, but Vigneau *et al.* [13] concluded that contrast media does not compromise the allograft’s function or survival. Embolization caused by instrumentation can be another complexity. Forbes *et al.* [14] suggest inserting the device through the side contralateral to the transplantation site to minimize the risk of injury and embolization in allograft artery. EVAR entails thorough follow-up to evaluate for endoleak and device function [15].

In conclusion, it seems that the transplanted population requires a careful follow-up because aneurysms tend to have an aggressive natural course in these patients. Although the choice of management should be individualized as each method has its limitations, endovascular repair seems a safer and more feasible alternative in patients with a history of transplantation.

## Supplementary Material

Media1_rjab332

media2_rjab332
